# Supplementary material for: Dual targeting of PI3K and FGFR or CDK4/6 reveals synergistic responses in HPV-positive and HPV-negative oropharyngeal cancer spheroids
Source: Mol Ther Oncol. 2026 Jun 30;34(3):201283. doi: 10.1016/j.omton.2026.201283 (PMC13380164; doi:10.1016/j.omton.2026.201283)
Supplement: Document S1. Figures S1 and S2 [file mmc1.pdf]

OMTON, Volume *34*

Supplemental information

Dual targeting of PI3K and FGFR or CDK4/6 reveals synergistic responses in HPV-positive and HPV-negative oropharyngeal cancer spheroids

Madeleine Birgersson, Monika Lukoseviciute, Ourania N. Kostopoulou, and Tina Dalianis

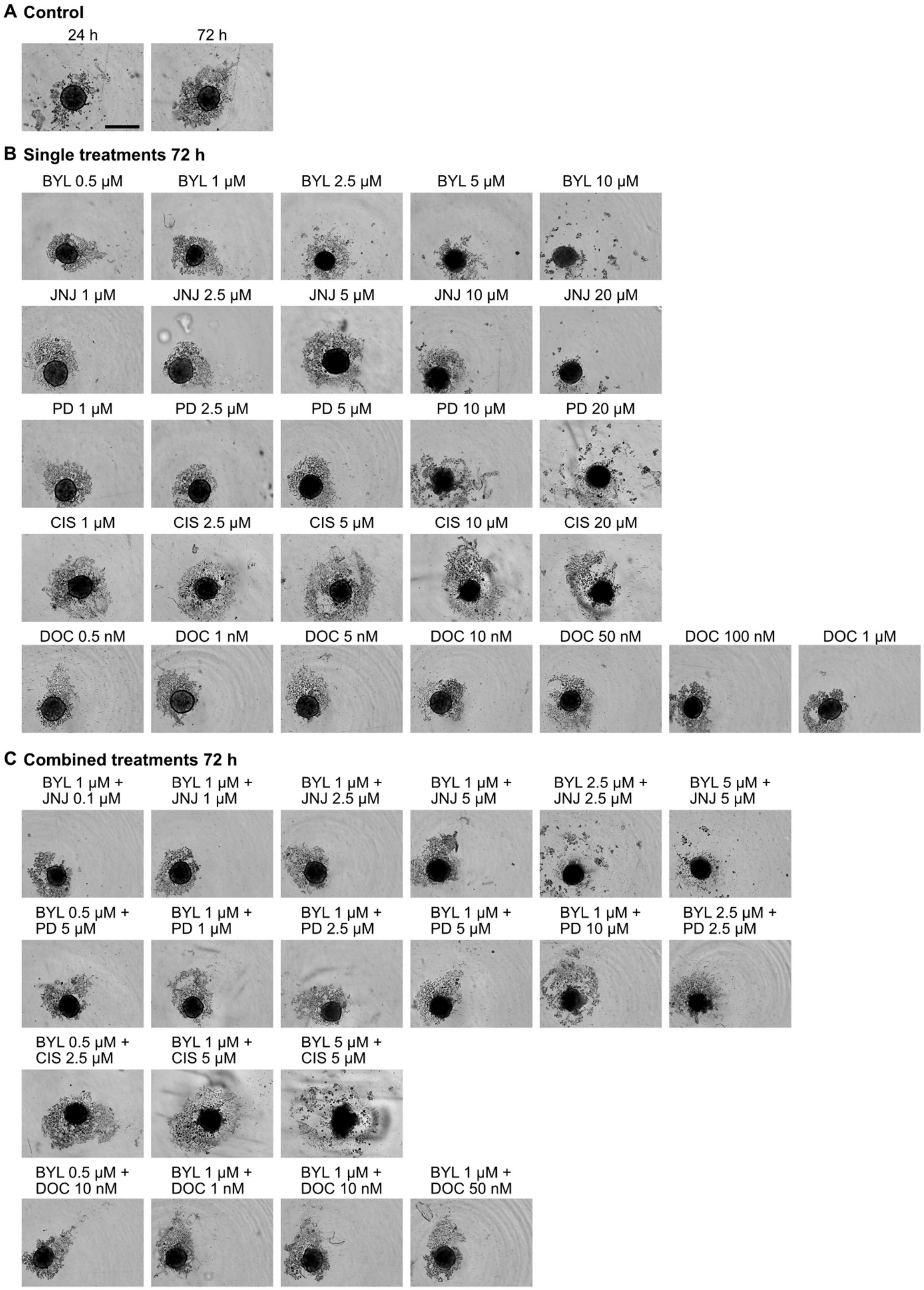

Figure S1. Effects of single PI3K, FGFR and CDK4/6 inhibitors and chemotherapeutic agents, and their combination with a PI3K inhibitor, on CU-OP-20 spheroid growth. Spheroid growth was monitored using the

Incucyte® SX5 Live-Cell Analysis Instrument from Sartorius. Representative images at 72h after treatment with: (A) PBS (control), (B) single treatment with BYL719, JNJ-42756493, PD-0332991, cisplatin, docetaxel, and (C) their combination with BYL719. The scale bar represents 400 µm.

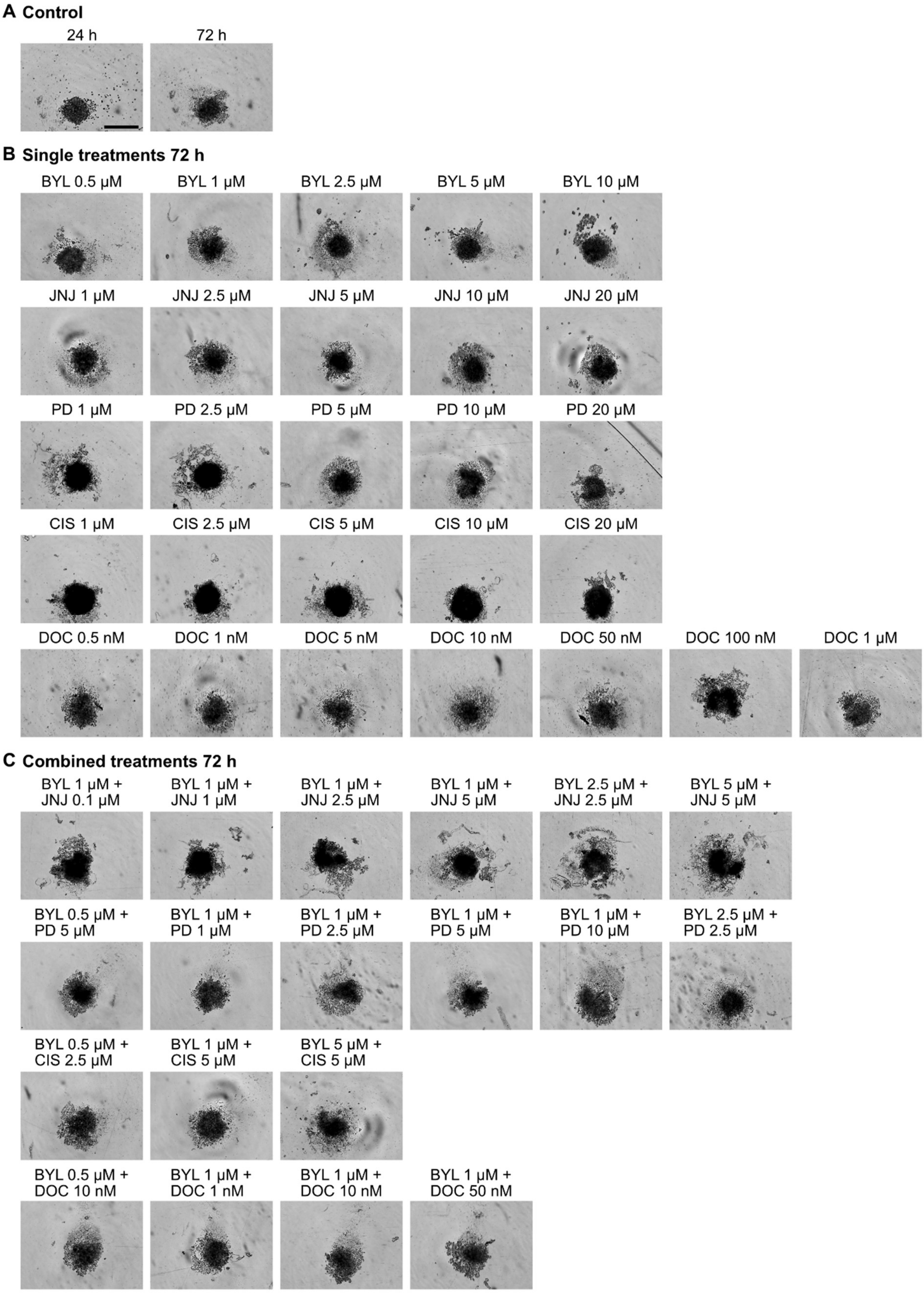

Figure S2. Effects of single PI3K, FGFR and CDK4/6 inhibitors and chemotherapeutic agents, and their combination with a PI3K inhibitor, on CU-OP-17 spheroid growth. Spheroid growth was monitored using

the Incucyte® SX5 Live-Cell Analysis Instrument from Sartorius. Representative images at 72h after treatment with: (A) PBS (control), (B) single treatment with BYL719, JNJ-42756493, PD-0332991, cisplatin, docetaxel, and (C) their combination with BYL719. The scale bar represents 400 μm.
