# Supplementary material for: Dual targeting of PI3K and FGFR or CDK4/6 reveals synergistic responses in HPV-positive and HPV-negative oropharyngeal cancer spheroids
Source: Mol Ther Oncol. 2026 Jun 30;34(3):201283. doi: 10.1016/j.omton.2026.201283 (PMC13380164; doi:10.1016/j.omton.2026.201283)
Supplement: Document S2. Article plus supplemental information [file mmc2.pdf]

Molecular Therapy
Oncology
Original Article

Dual targeting of PI3K and FGFR or CDK4/6 reveals synergistic responses in HPV-positive and HPV-negative oropharyngeal cancer spheroids

Madeleine Birgersson,[1,2] Monika Lukoseviciute,[1] Ourania N. Kostopoulou,[1,2] and Tina Dalianis[1,2]

[1]Department of Oncology-Pathology, Karolinska Institutet, 171 64 Stockholm, Sweden; [2]Medical Unit Head-, Neck-, Lung- and Skin Cancer Theme Cancer, Karolinska University Hospital, 171 64 Stockholm, Sweden

Human papillomavirus-positive (HPV$^+$) oropharyngeal squamous cell carcinoma (OPSCC) generally has a better prognosis than HPV-negative (HPV$^-$) OPSCC. Still, chemoradiotherapy does not cure all patients; therefore, novel treatments are needed. In monolayers of HPV$^{+/-}$ OPSCC CU-OP cell lines, we previously demonstrated that phosphoinositide 3-kinase (PI3K), fibroblast growth factor receptor (FGFR), and cyclin-dependent kinase 4/6 (CDK4/6) inhibitors reduced viability and had synergistic effects when combined. Here, to better simulate the *in vivo* state, the effects of these inhibitors and cytostatics were assessed in spheroids of HPV$^{+/-}$ CU-OP cell lines. All spheroids were treated with single administrations of PI3K (BYL719), FGFR (JNJ-42756493), or CDK4/6 (PD-0332991) inhibitors or cisplatin or docetaxel, as well as with combinations of BYL719 with all named drugs. All inhibitors reduced cell viability and growth in all spheroids, especially in CU-OP-2, while the effects of cisplatin and docetaxel were more limited. Combining BYL719 with all other drugs resulted in additive or synergistic effects in CU-OP-2, while corresponding treatments generally required higher doses to reach similar effects in the other cell lines. Interestingly, the high drug sensitivity of CU-OP-2 differed from the observations made in earlier monolayer studies, where CU-OP-20 was the most sensitive cell line, highlighting the importance of using spheroids in drug screenings.

INTRODUCTION

Human papillomavirus-positive (HPV$^+$) oropharyngeal squamous cell carcinoma (OPSCC), especially tonsillar and base-of-tongue cancer, its two major subsites, have increased in incidence and have better clinical outcomes than the corresponding HPV-negative (HPV$^-$) cancers.[1–10] Current chemoradiotherapy and, when needed, a neck dissection for removal of potential lymph node metastases, are associated with severe side effects, such as difficulties in swallowing, speaking, and neck stiffness. Still, these treatments do not cure all patients with HPV$^+$ cancer.[9,10]

To provide personalized therapy or novel curative options, studies on prognostic and targetable biomarkers in HPV$^+$ OPSCC have been pursued to identify specific molecular targets and potentially enable de-escalation of therapy.[11–15] Identified genetic mutations included, for e.g., phosphatidyl-inositol-4,5-bisphosphate 3-kinase catalytic subunit alpha (*PIK3CA*) and fibroblast growth factor receptor 3 (*FGFR3*).[11–15] Notably, inhibitors targeting these factors have been approved by the Food and Drug Administration (FDA) for clinical use in breast and urothelial cancers with respective *PIK3CA* or *FGFR3* mutations/translocations.[16,17] We have, therefore, tested phosphoinositide 3-kinase (PI3K) and FGFR inhibitors together with a cyclin-dependent kinase 4/6 (CDK4/6) inhibitor and have observed dose-dependent effects in monolayer (2D) OPSCC cell cultures. When combining two inhibitors or the inhibitors with the chemotherapeutic agents cisplatin and docetaxel, often used for OPSCC therapy, additive or synergistic effects were observed.[18,19]

Subsequently, in further studies aimed at better mimicking *in vivo* conditions, the PI3K inhibitor was evaluated in a single three-dimensional (3D) spheroid model harboring *PIK3CA* and *FGFR* mutations, in which a delayed reduction in viability was observed.[20] Moreover, the effect of fibroblast supernatant on the response to the PI3K, FGFR, and CDK4/6 inhibitors was explored, where the addition of fibroblast supernatant required higher drug doses to achieve comparable effects.[21]

Here, the impact of the PI3K, FGFR, and CDK4/6 inhibitors as well as the chemotherapeutic agents cisplatin and docetaxel and their combination was further scrutinized in spheroid cultures of HPV$^{+/-}$ OPSCC cell lines.

Received 15 April 2026; accepted 26 June 2026;
https://doi.org/10.1016/j.omton.2026.201283.

Correspondence: Madeleine Birgersson, Department of Oncology-Pathology, Karolinska Institutet, 171 64 Stockholm, Sweden.
E-mail: madeleine.birgersson@ki.se
Correspondence: Tina Dalianis, Department of Oncology-Pathology, Karolinska Institutet, 171 64 Stockholm, Sweden.
E-mail: tina.dalianis@ki.se

Published by Elsevier Inc. on behalf of The American Society of Gene and Cell Therapy.
This is an open access article under the CC BY license (http://creativecommons.org/licenses/by/4.0/).

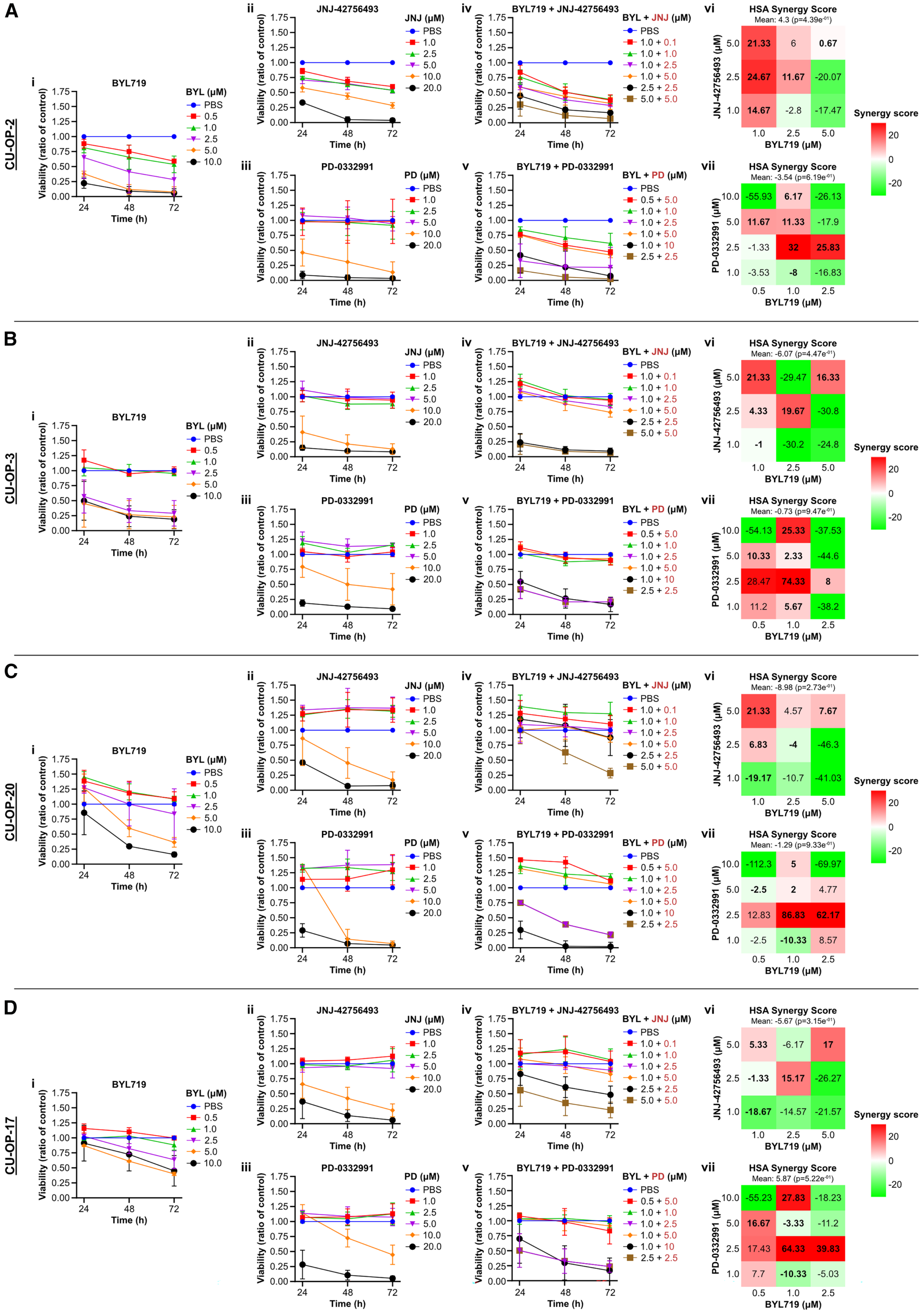

(legend on next page)

RESULTS

Effects of single and combined administrations of PI3K, FGFR, and CDK4/6 inhibitors and chemotherapeutic agents on OPSCC spheroid viability

HPV$^+$ CU-OP-2, -3, and -20 and HPV$^-$ CU-OP-17 spheroids were exposed to single and combined treatments for 72 h. The data for each treatment are presented in the following text, and significance has been evaluated in comparison to the corresponding PBS control (Figures 1 and 2).

(1) BYL719: HPV$^+$ CU-OP-2 and -3, especially CU-OP-2 with an *FGFR3* and a *PIK3CA* mutation, were generally more sensitive to BYL719 than CU-OP-20 and -17 (Figures 1A–1Di). Nevertheless, all CU-OP spheroids showed a significant decrease in cell viability with the two highest doses (5 and 10 μM) at 72 h ($p < 0.05$).

(2) JNJ-42756493: HPV$^+$ CU-OP-2 was more sensitive to JNJ-42756493 than the other CU-OP lines. However, all remaining spheroids showed a significant response to the two highest doses (10 and 20 μM) of the drug at all time points ($p < 0.05$) (Figures 1A–1Dii).

(3) PD-0332991: all CU-OP spheroids responded significantly only to the two highest doses (10 and 20 μM) of PD-0332991 at 72 h ($p < 0.0001$) (Figures 1A–1Diii).

(4) Cisplatin: only CU-OP-2 spheroids displayed a significant response to cisplatin, with a decrease in viability at 72 h with all doses ($p < 0.05$) (Figures 2A–2Dii).

(5) Docetaxel: CU-OP-2 spheroids were the most sensitive to docetaxel, and a significant decrease in viability was obtained with the highest doses (0.05-1 μM) at 72 h ($p < 0.05$), while the other cell lines responded only to the highest dose (1 μM) ($p < 0.001$) (Figures 2A–2Diii).

(6) BYL719 and JNJ-42756493: combining BYL719 and JNJ-42756493 showed mainly synergistic effects on HPV$^+$ CU-OP-2, especially 72 h after treatment, where all drug combinations significantly decreased cell viability ($p < 0.01$) (Figures 1Aiv and 1Avi). The same combination also showed tendencies of corresponding additive/synergistic effects in the remaining CU-OP spheroids. However, most doses did not sufficiently inhibit viability, as a significant reduction in viability was observed only with the highest doses for the other cell lines after 72 h (Figures 1B–1Div and 1Dvi).

(7) BYL719 and PD-0332991: combining BYL719 and PD-0332991 had some additive and synergistic effects on the decrease of viability on all CU-OP spheroids. This was especially true for HPV$^+$ CU-OP-2, where statistically significant inhibitory effects were demonstrated for all drug combinations 72 h after treatment ($p < 0.05$) (Figures 1Av and 1Avii, respectively). For HPV$^+$ CU-OP-3 and -20, and HPV$^-$ CU-OP-17, half of the combinations (1 μM BYL719 + 2.5 μM PD-0332991, 1 μM BYL719 + 10 μM PD-0332991, and 2.5 μM BYL719 + 2.5 μM PD-0332991) were also effective at significantly inhibiting viability ($p < 0.0001$) (Figures 1B–1Dv and 1Dvii).

(8) BYL719 and cisplatin: the BYL719 and cisplatin combination elicited mainly synergistic effects in all spheroid cell lines except CU-OP-20, where mainly additive effects were observed (Figures 2A–2Div and 2Dvi). The most effective combination was adding 5 μM BYL719 to 5 μM cisplatin, where additive interactions were demonstrated in all CU-OP spheroids at 72 h ($p < 0.0001$) (Figures 2A–2Div and 2Dvi).

(9) BYL719 and docetaxel: the BYL719 and docetaxel combination efficiently elicited only synergistic effects with a significant decrease of viability in HPV$^+$ CU-OP-2 after 72 h of treatment with all doses ($p < 0.0001$), while the viability of the remaining spheroids was not affected (Figures 2A–2Dv and 2Dvii).

In summary, upon combining the inhibitors, additive synergistic effects were uncovered, especially in the HPV$^+$ CU-OP-2 cell line. Although the combination of BYL719 with the other inhibitors or with cisplatin or docetaxel resulted in additive and synergistic effects in the other spheroids, their combinations were rarely effective.

Effects of PI3K, FGFR, and CDK4/6 inhibitors and cisplatin and docetaxel on HPV$^+$/HPV$^-$ CU-OP spheroid growth

The effects on spheroid growth after different drug treatments were also investigated. Due to individual changes in the spheroid structure of the different CU-OP cell lines after treatment, including large halo structures, exact measurements could not be recorded, and a non-quantitative assessment was performed instead. In the following text, details for each cell line up to 72 h after the various treatments are presented separately (Figures 3 and 4; Figures S1, and S2).

(1) CU-OP-2: compared to the control, which showed growth and an intact spheroid ring, all inhibitors and cytostatic drugs and their combinations affected growth and dispersed the intact spheroid ring at intermediate to high doses (Figures 3A and 3B). Moreover, all intermediate to high doses led to an increased halo-like structure indicative of cellular shedding, suggesting cell dissociation from the main spheroid body (Figure 3B). Furthermore, all combinations induced dispersion of the intact spheroid ring and an increased halo-like structure with lower doses (Figure 3C).

(2) CU-OP-3: none of the single and combined treatments affected the spheroid size. However, spheroid dispersal was

Figure 1. Effects of single and combined inhibitors targeting PI3K, FGFR, and CDK4/6 on the viability of CU-OP spheroids

For each cell line, (A) CU-OP-2, (B) CU-OP-3, (C) CU-OP-20, and (D) CU-OP-17, subimages (i)–(v) show viability following treatment with the indicated single/combined agents (i, BYL719; ii, JNJ-42756493; iii, PD-0332991; iv, BYL719 + JNJ-42756493; and v, BYL719 + PD-0332991). Subimages (vi) and (vii) display synergy analyses for the combinations BYL719 + JNJ-42756493 (vi) and BYL719 + PD-0332991 (vii). The data is presented as mean ± SD.

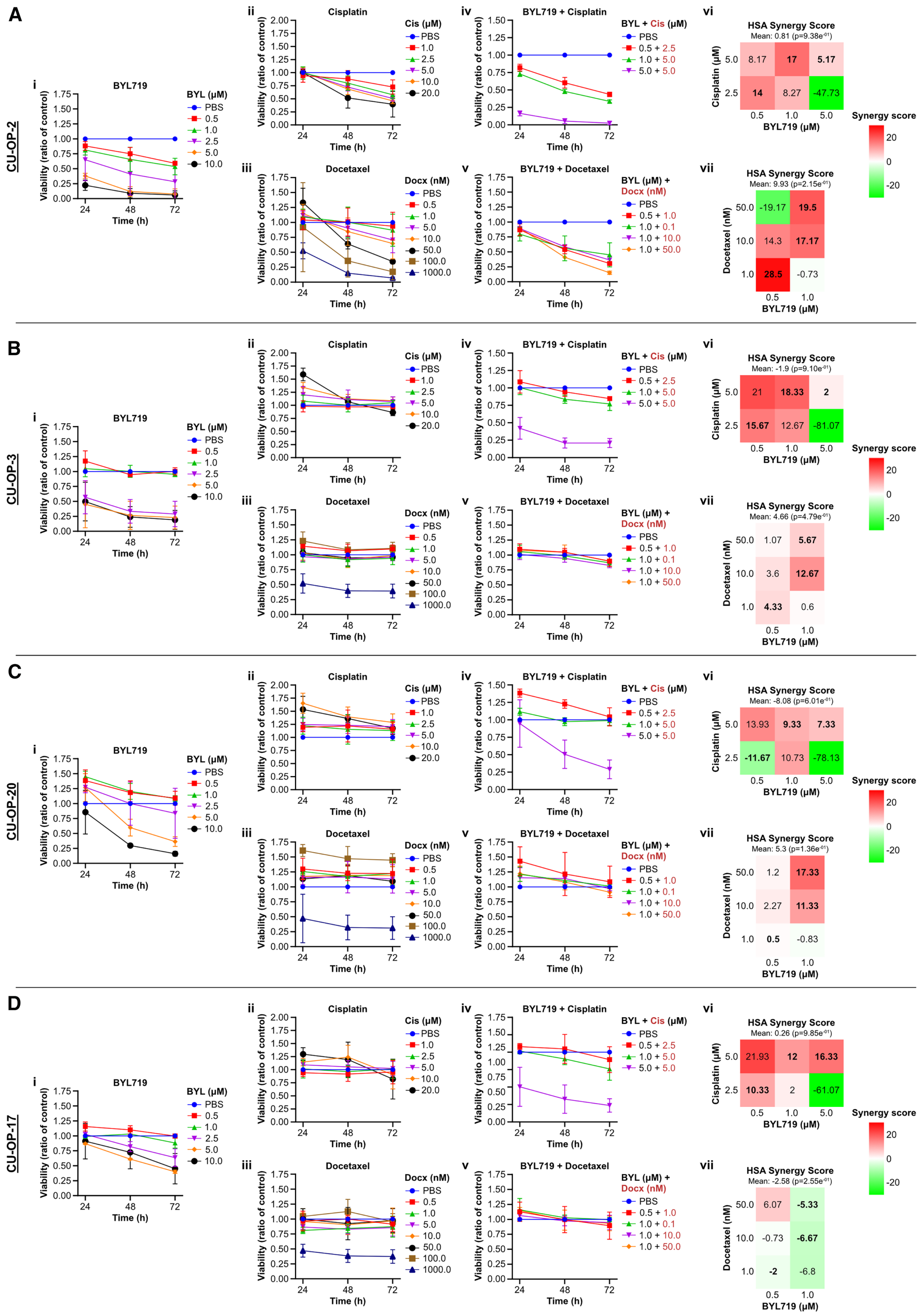

(legend on next page)

induced, especially with the single and combined inhibitor administrations (Figure 4).

(3) CU-OP-20: the highest doses of all single drug treatments inhibited spheroid growth compared with the control (Figures S1A and S1B). Moreover, combining BYL719 with PD-0332991 or cisplatin destroyed the spheroid structure significantly (Figure S1C).

(4) CU-OP-17: the effects of the single inhibitors on CU-OP-17 spheroid size and growth were modest, while the cytostatics considerably disrupted spheroid structure (Figure S2). Similarly, all combinational treatments greatly affected the spheroid structure.

In summary, the effects of the single and combined treatments on spheroid growth were in concordance with the viability data and displayed overall the greatest effects on inhibition of growth in CU-OP-2.

Effects of PI3K, FGFR, and CDK4/6 inhibitors on HPV$^+$ CU-OP-2 and -3 spheroid migration

The effects of single and combined administrations of all the inhibitors on HPV$^+$ CU-OP-2 and -3 spheroid migration were also assessed. However, no significant migration was observed for CU-OP-3, and the inhibitors' effect on migration could therefore not be assessed.

In CU-OP-2, all single inhibitors reduced the spheroid migration to some extent (Figure 5). The migration of CU-OP-2 was already notably inhibited with 0.5 μM BYL719 and completely inhibited with 2.5 μM BYL719, whereas the same inhibition was obtained at considerably higher concentrations of JNJ-42756493 and PD-0332991. JNJ-42756493 demonstrated inhibition of migration at 1 μM and complete inhibition at 10 μM. Meanwhile, PD-0332991 had considerably less impact on migration, with significant inhibition at 2.5 μM, but complete inhibition was observed only at 20 μM. However, despite a slightly enhanced inhibition of migration with the combination of 1 μM BYL719 + 0.1 μM JNJ-42756493, neither of the inhibitor combinations resulted in a significantly increased inhibition of migration compared with the single administrations.

Effects of PI3K, FGFR, and CDK4/6 inhibitors on proliferation and apoptosis in HPV$^+$ CU-OP-2 and -3 spheroids

To assess how the inhibitors affect proliferation and apoptosis, HPV$^+$ CU-OP-2 and -3 spheroids were stained for the proliferation marker Ki-67 and the apoptotic marker cleaved caspase-3. The data are illustrated in Figure 6.

Firstly, in the untreated spheroids, the Ki-67 staining showed a markedly higher proportion of proliferating cells in the CU-OP-2 spheroids than in the CU-OP-3 spheroids, while there appears to be no difference in the ratio of cleaved caspase-3 positive cells (Figures 6A and 6B). In CU-OP-2, 0.5 μM of BYL719 significantly reduced the size of the spheroid, with a slight decrease in the ratio of Ki-67 positive cells and an increase in cleaved caspase-3 positive cells (Figure 6A). While 5 μM of PD-0332991 did not result in an equal reduction in spheroid size, and 1 μM of JNJ-42756493 did not reduce the size at all, there was still a clear reduction in the fraction of proliferative cells with both single drug administrations (Figure 6A). However, the single 1 μM BYL719 treatment and the two combination treatments resulted in too loose CU-OP-2 spheroids, which could not be properly embedded, as can be seen in the BYL719 and PD-0332991 cleaved caspase-3 staining (Figure 6A). The effects on proliferation and apoptosis could, therefore, not be assessed for these treatments.

In contrast to CU-OP-2, the CU-OP-3 spheroids appeared to have increased slightly in size after single treatment administrations, most likely due to looser spheroids, and an increased Ki-67 staining was especially noticeable after treatment with 0.5 μM BYL719 and 1 μM JNJ-42756493 (Figure 6B). Nevertheless, all single treatments, and especially the two combinations 0.5 μM BYL719 + 5 μM PD-0332991 and 1 μM BYL719 + 1 μM JNJ-42756493, resulted in an increased expression of cleaved caspase-3 in the CU-OP-3 spheroids (Figure 6B).

DISCUSSION

To better align with the *in vivo* state and compare with earlier data in monolayer cultures, spheroids of HPV$^+$/HPV$^-$ OPSCC cell lines with different mutational profiles were exposed to single or combined PI3K, FGFR, and CDK4/6 inhibitors and cisplatin and docetaxel. Their effects on cell viability and growth were monitored in all cell lines, and potential effects on migration and expression of Ki-67 and cleaved caspase-3 were assessed in spheroids of the treatment-sensitive CU-OP-2 with *PIK3CA* and *FGFR3* mutations and the treatment-resistant CU-OP-3 with corresponding wild-type genes.

With the doses used, all three single inhibitors BYL719, JNJ-42756493, and PD-0332991 were shown to reduce viability in all spheroids to some extent, especially with the highest doses, while the effects of the chemotherapeutic agents were modest at the doses applied. Moreover, combining BYL719 with JNJ-42756493, PD-0332991, or cisplatin generally showed additive or synergistic effects. The combinations were especially efficient in CU-OP-2 spheroids, where most doses resulted in additive or synergistic effects and efficient viability reduction, while only the higher doses gave the same effect on the other spheroids. Although spheroid growth was more difficult to assess, possibly due to differences in spheroid appearance and structure between the CU-OP cells, it was consistent with the viability data.

Figure 2. Effects of single chemotherapeutic agents and their combination with a PI3K inhibitor on the viability of CU-OP spheroids

For each cell line, (A) CU-OP-2, (B) CU-OP-3, (C) CU-OP-20, and (D) CU-OP-17, subimages (i)–(v) show viability following treatment with the indicated single/combined agents (i, BYL719; ii, cisplatin; iii, docetaxel; iv, BYL719 + cisplatin; and v, BYL719 + docetaxel). Subimages (vi) and (vii) display synergy analyses for the combinations BYL719 + cisplatin (vi) and BYL719 + docetaxel (vii). The data is presented as mean ± SD.

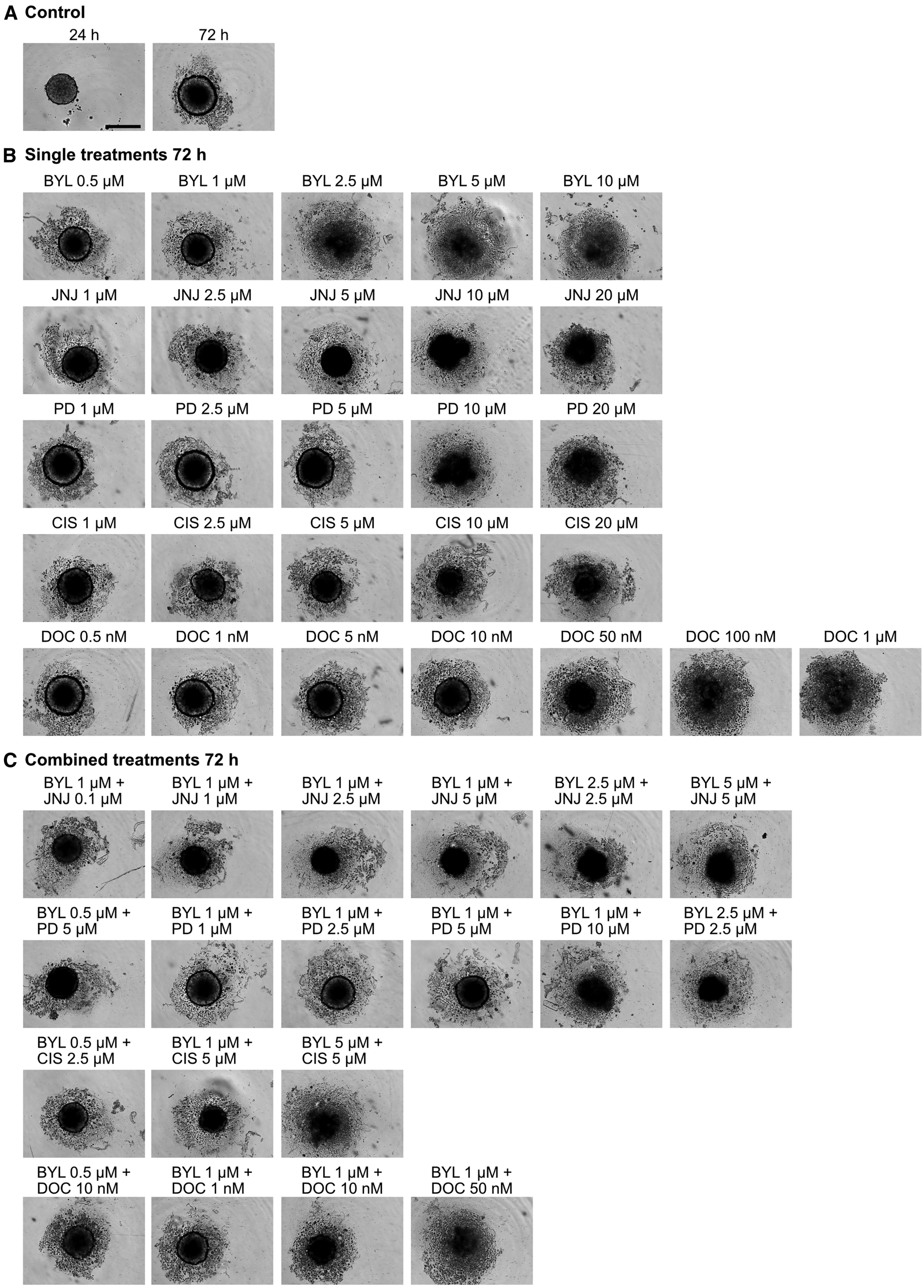

(legend on next page)

In addition to viability and spheroid growth, the inhibitors' effect on spheroid migration and expression of proliferation and apoptosis markers were assessed in CU-OP-2 and -3. While migration could only be assessed in CU-OP-2 due to a lack of migration by the CU-OP-3 spheroids, the results closely mirrored the viability data, further validating the impact of the drugs.

Comparing these data to earlier monolayer studies,[18,19] there are many similarities but also several intriguing differences. Firstly, CU-OP spheroids were overall more sensitive to PD-0332991 than corresponding monolayer cultures. However, while CU-OP-2 spheroids were the most sensitive of the CU-OP spheroids, CU-OP-2 was the least sensitive cell line in the monolayer setting.[19] In contrast, all CU-OP spheroids, except for CU-OP-2, displayed a reduced sensitivity to BYL719 compared with monolayer cultures.[18]

Notably, one of the most intriguing differences is that in the monolayer cultures, although all cell lines were sensitive to BYL719 to some extent, CU-OP-20 tended to be the most sensitive cell line and was more responsive than CU-OP-2,[18] which was not observed in the current study with spheroids. Instead, the PI3K and FGFR inhibitors BYL719 and JNJ-42756493, respectively, showed a particularly strong reduction of viability in CU-OP-2 spheroids harboring *PIK3CA* and *FGFR3* mutations. The high sensitivity of CU-OP-2 to BYL719 could be expected due to the *PIK3CA* mutation in CU-OP-2. However, as CU-OP-20 also harbors a *PIK3CA* mutation, a corresponding response was anticipated, but this was not the case. The reason for this discrepancy is currently not known and has yet to be extensively explored. One possibility is that the additional *FGFR3* mutation that CU-OP-2 harbors sensitizes this cell line to the effect of the BYL719 inhibitor in the spheroid context.

The difference in drug sensitivity between monolayers and spheroids is intriguing and could be explained by several factors. Firstly, in this study, the cell lines were grown without fibroblast feeders, and we have previously shown that fibroblast-secreted factors reduce the drug sensitivity of the cells.[21] However, the context of being cultured as spheroids may contribute to additional factors, such as an oxygen/nutrient gradient and increased cell-cell interaction,[22,23] which may affect drug responses. Spheroid cultures can also induce changes in gene expression compared with when cultured as monolayers. In long-time cultures, spheroids have been shown to exhibit higher expression of genes related to cell-cell junctions and stemness and may exhibit negative regulation of proliferation, while monolayers show increased expression of genes involved in cell-cycle progression and extracellular matrix (ECM) production.[24]

In support of this, a recent study in a sonic hedgehog (SHH) medulloblastoma stem cell model showed that spheroid cultures were more sensitive to JNJ-42756493 than monolayer cultures.[25] Thus, it could be speculated that spheroid formation is particularly sensitive to FGFR inhibitors, but additional experimental data are needed.

There are several limitations in this study, with one of the main limitations being the difference in spheroid formation between the four cell lines. Although the spheroids were formed through the same conditions, except for higher cell seeding for CU-OP-17, the differences, e.g., in appearance, add to the variability of the different assessed factors. On top of that, despite the known HPV and mutational status of the cell lines, their transcriptomic profiles and how they change in spheroid cultures are currently unknown. Such data would be highly valuable to obtain a mechanistic understanding of the drug responses and are, therefore, planned for future studies. Nevertheless, the use of spheroid cell cultures, as shown here, could still enable a high-throughput preliminary faster drug screening prior to embarking on using xenografts or organoid models for more detailed studies.

To conclude, while there were consistencies in the treatment response of CU-OP cells grown as monolayers and spheroids, spheroid cultures induced a higher variability between the cell lines, where CU-OP-2 was the most sensitive to treatment. While further studies are needed to get a mechanistic understanding of these differences, this study highlights the importance of including spheroids in drug screening, as they better mimic *in vivo* conditions.

MATERIALS AND METHODS

Cell lines and seeding

OPSCC cell lines, HPV^{+} CU-OP-2 (with a *pE545K-PIK3CA* and a *pS249C-FGFR3* mutation), CU-OP-3, CU-OP-20 (with a *pE545K-PIK3CA* mutation), and HPV^{-} CU-OP-17, were initially provided by Dr. N. Powell, Cardiff University, UK, with further details and their adaptation to growth without feeders described before.[20,26,27]

All spheroids were generated in Thermo Scientific Nunclon Sphera 96-Well, Nunclon Sphera-Treated, U-Shaped-Bottom Microplates (Thermo Fisher, Stockholm, Sweden) as reported previously.[20,21] Seeding of 2,000 cells/well for all cell lines except CU-OP-17, which required 3,000 cells/well, in 100 μL Glasgow's Minimum Essential Medium (GMEM) attained a spheroid diameter of 300-400 μm after 72 h, at which time point treatment was administered for an additional 72 h.

Drugs and treatments

The PI3K, FGFR, and CDK4/6 inhibitors BYL719 (alpelisib), JNJ-42756493 (erdafitinib), and PD-0332991 (palbociclib), respectively, were purchased from TargetMol (San Diego, California, US) at a concentration of 10 mM in DMSO. Working dilutions

Figure 3. Effects of single PI3K, FGFR, and CDK4/6 inhibitors and chemotherapeutic agents and their combination with a PI3K inhibitor on CU-OP-2 spheroid growth

Representative images of spheroid growth at 72 h after treatment with: (A) PBS (control); (B) single treatment with BYL719 (BYL), JNJ-42756493 (JNJ), PD-0332991 (PD), cisplatin (CIS), and docetaxel (DOC); and (C) their combination with BYL719. The scale bars represent 400 μm.

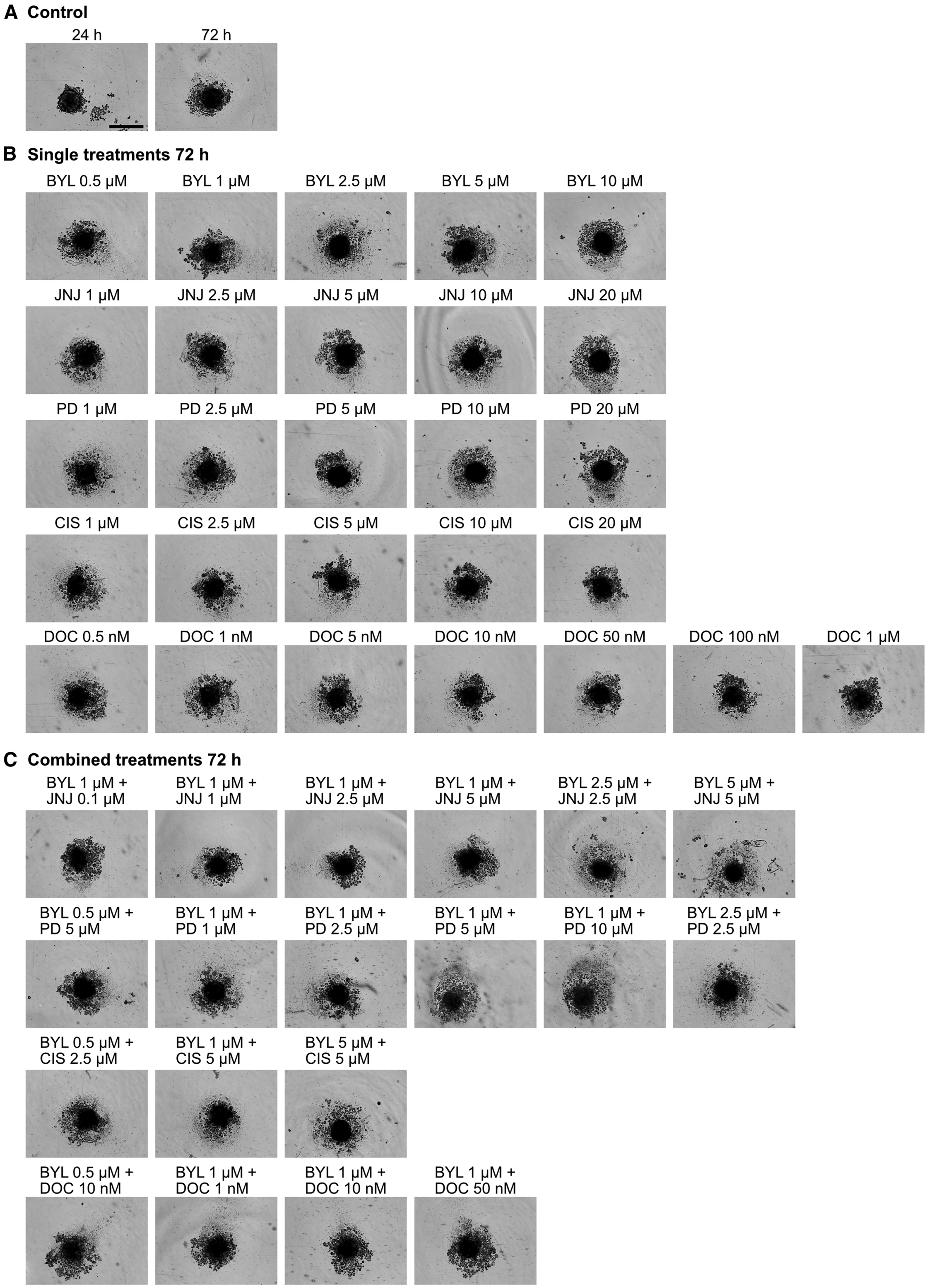

Figure 4. Effects of single PI3K, FGFR, and CDK4/6 inhibitors and chemotherapeutic agents and their combination with a PI3K inhibitor on CU-OP-3 spheroid growth

(legend continued on next page)

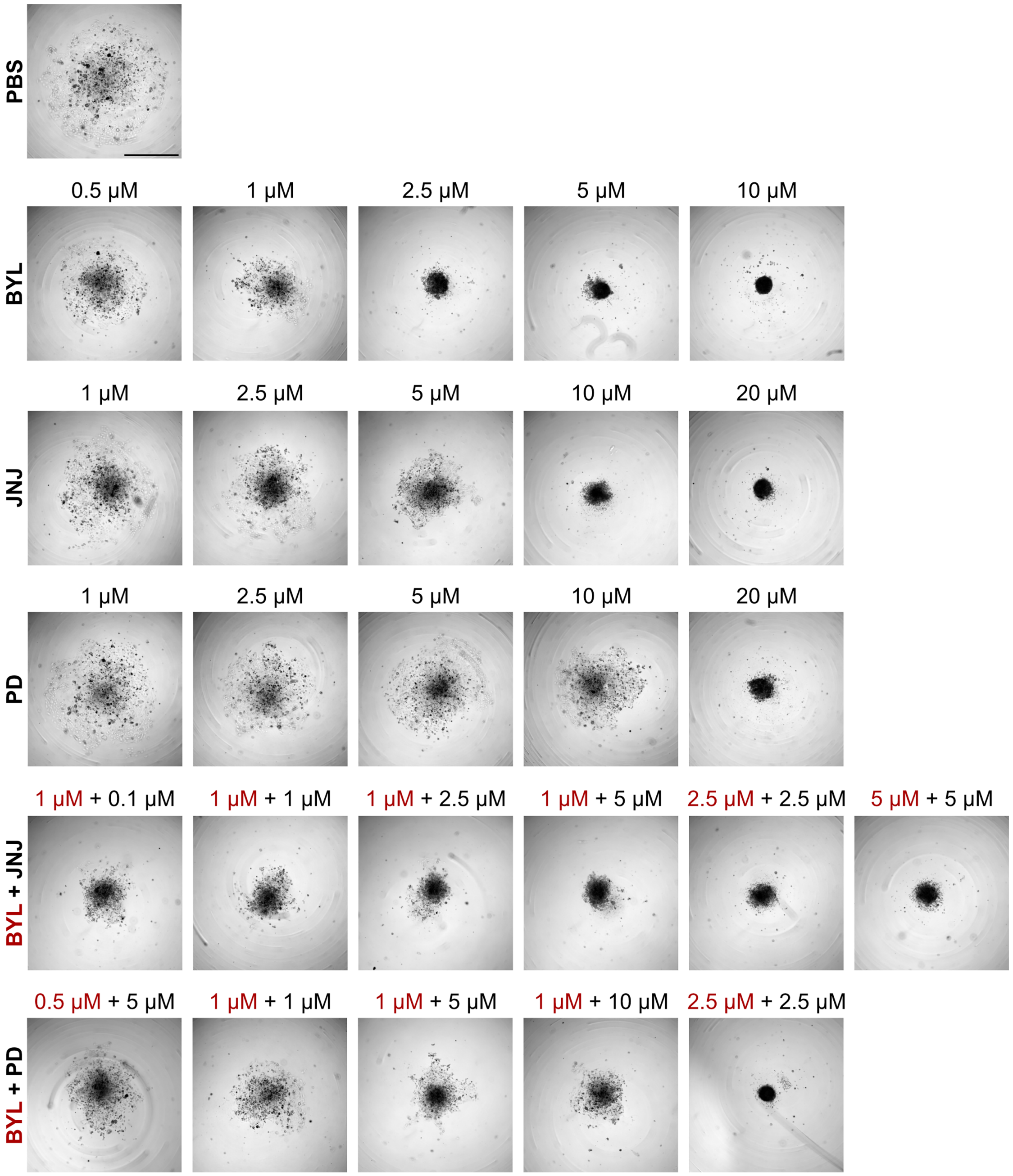

Figure 5. Effects of single PI3K, FGFR, and CDK4/6 inhibitors and their combination with a PI3K inhibitor on CU-OP-2 spheroid migration
Representative images of spheroid migration 72 h after treatment with PBS, single BYL719 (BYL), JNJ-42756493 (JNJ), and PD-0332991 (PD) or their combination with BYL719. The scale bars represent 1 mm.

(0.5-20 μM) were prepared in PBS. Cisplatin (1–20 μM) and docetaxel (0.5 nM-1 μM), acquired from Accord Healthcare Limited (Middlesex, UK), were diluted in PBS.

RealTime-Glo MT cell viability assay

Viability of spheroids was measured 24–72 h after treatment using RealTime-Glo MT Cell viability assay (Promega, Stockholm,

Representative images of spheroid growth at 72 h after treatment with: (A) PBS (control); (B) single treatment with BYL719 (BYL), JNJ-42756493 (JNJ), PD-0332991 (PD), cisplatin (CIS), and docetaxel (DOC); and (C) their combination with BYL719. The scale bars represent 400 μm.

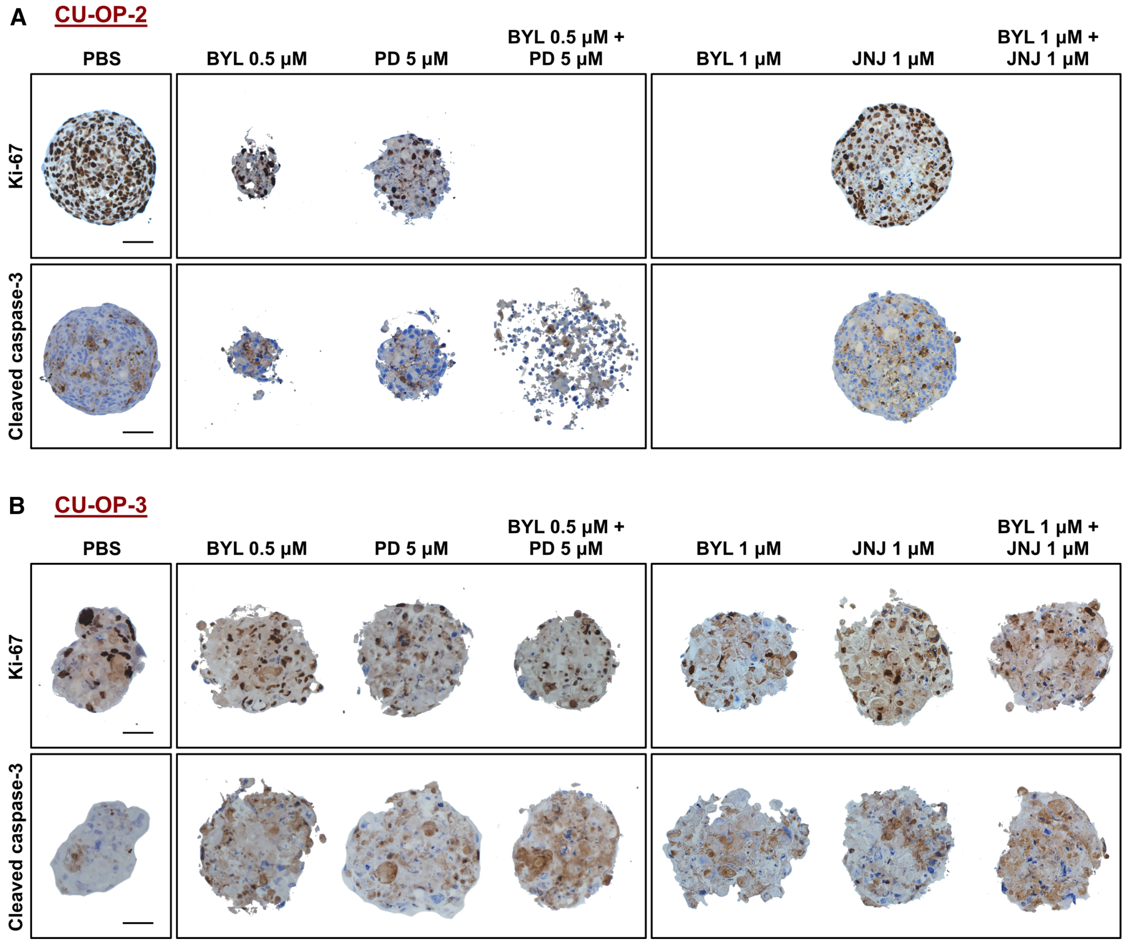

Figure 6. Effects of single PI3K, FGFR, and CDK4/6 inhibitors and their combination with a PI3K inhibitor on proliferation and apoptosis in CU-OP-2 and -3
IHC staining of Ki-67 and cleaved caspase-3 in (A) CU-OP-2 and (B) CU-OP-3 spheroids collected 72 h after treatment with PBS, single BYL719 (BYL), JNJ-42756493 (JNJ), and PD-0332991 (PD) or their combination with BYL719. The scale bars represent 100 µm.

Sweden) according to the manufacturer's instructions and as described before.[21]

Spheroid size during treatment

Real-time imaging was captured using the Incucyte SX5 Live-Cell Analysis Instrument from Sartorius (Sartorius, Essen Bioscience, Göttingen, Germany) as described before.[21]

Spheroid migration

U-bottom plates were coated in gelatin by adding 100 µL 0.2% gelatin solution (3-H Biomedical, Uppsala, Sweden) and incubating for 30 min. After removing the excess gelatin, pre-formed spheroids (see aforementioned details) were transferred to the coated plates and treatment was added. Images were captured using a Leica DMi8 microscope (Leica Microsystems, Wetzlar, Germany) at 72 h post-treatment.

Immunohistochemical evaluation of proliferation and apoptosis markers

72 h after treatment, spheroids from a 96-well spheroid plate/condition were fixed, embedded, and stained as previously described,[25] using primary antibodies anti-Ki67 (1:800, DAKO, #A0047, RRID: AB_2314699, UK Ltd., Cambridgeshire, UK) and anti-cleaved caspase-3 (anti-CC-3) (1:2000, Cell Signaling, #9661, RRID: AB_2341188, Danvers, MA, USA). Images were obtained using the Olympus BH-2 microscope (Tokyo, Japan) and DMK 33UX264 series monochrome industrial camera (The Imaging Source, Bremen, Germany).

Evaluation of the effectiveness of various drug combinations

To evaluate the efficacy of the drug combinations, the Synergy Finder Plus platform (https://synergyfinderplus.org/) was utilized, and the highest single agent (HSA) model was applied. According to this approach, an HSA score above 10 denotes drug synergy, scores

between −10 and 10 indicate additive interactions, and scores below −10 suggest antagonistic effects.[28]

Statistical analysis

Two-way ANOVA, followed by Dunnett's multiple comparisons test, was used to verify the effect of the single inhibitors and chemotherapeutic agents and their combination compared with the PBS control.

DATA AND CODE AVAILABILITY

All data generated or analyzed during this study are included in this published article and its supplemental information.

ACKNOWLEDGMENTS

We thank Dr. Neil Powell, Cardiff University, UK, for kindly providing us with the cell lines. This research was funded by the Swedish Cancer Foundation (grant no. 23 2700), the Stockholm Cancer Society (grant no. 241131), the Lars Hierta Memorial Foundation (grant no FO2024-0399), the Sigurd och Elsa Goljes Memorial Foundation (grant no LA2025-0024), the Stockholm City Council, and Karolinska Institutet.

AUTHOR CONTRIBUTIONS

Conceptualization, M.B., O.N.K., and T.D.; methodology, M.B.; software, M.B. and M.L.; validation, M.B., O.N.K., and T.D.; formal analysis, M.B., M.L., and T.D.; investigation, M.B., O.K., and T.D.; resources, M.B., O.N.K., and T.D.; data curation, M.B. and T.D.; writing - original draft preparation, M.B. and T.D.; writing - review and editing, M.B., O.N.K., M.L., and T.D.; visualization, M.B.; supervision, M.B., O.N.K., and T.D.; project administration, M.B. and T.D.; funding acquisition, M.B., O.N.K., and T.D. All authors have read and agreed to the submitted version of the manuscript.

DECLARATION OF INTERESTS

The authors declare no competing interests.

SUPPLEMENTAL INFORMATION

Supplemental information can be found online at https://doi.org/10.1016/j.omton.2026.201283.

REFERENCES

1. Gillison, M.L., Koch, W.M., Capone, R.B., Spafford, M., Westra, W.H., Wu, L., Zahurak, M.L., Daniel, R.W., Viglione, M., Symer, D.E., et al. (2000). Evidence for a Causal Association Between Human Papillomavirus and a Subset of Head and Neck Cancers. J. Natl. Cancer Inst. *92*, 709–720.

2. Mellin, H., Friesland, S., Lewensohn, R., Dalianis, T., and Munck-Wikland, E. (2000). Human papillomavirus (HPV) DNA in tonsillar cancer: Clinical correlates, risk of relapse, and survival. Int. J. Cancer *89*, 300–304.

3. Dahlgren, L., Dahlstrand, H.M., Lindquist, D., Högmo, A., Björnestål, L., Lindholm, J., Lundberg, B., Dalianis, T., and Munck-Wikland, E. (2004). Human papillomavirus is more common in base of tongue than in mobile tongue cancer and is a favorable prognostic factor in base of tongue cancer patients. Int. J. Cancer *112*, 1015–1019.

4. Lindquist, D., Romanitan, M., Hammarstedt, L., Näsman, A., Dahlstrand, H., Lindholm, J., Onelöv, L., Ramqvist, T., Ye, W., Munck-Wikland, E., and Dalianis, T. (2007). Human papillomavirus is a favourable prognostic factor in tonsillar cancer and its oncogenic role is supported by the expression of E6 and E7. Mol. Oncol. *1*, 350–355.

5. Näsman, A., Attner, P., Hammarstedt, L., Du, J., Eriksson, M., Giraud, G., Ährlund-Richter, S., Marklund, L., Romanitan, M., Lindquist, D., et al. (2009). Incidence of human papillomavirus (HPV) positive tonsillar carcinoma in Stockholm, Sweden: An epidemic of viral-induced carcinoma? Int. J. Cancer *125*, 362–366.

6. Chaturvedi, A.K., Engels, E.A., Pfeiffer, R.M., Hernandez, B.Y., Xiao, W., Kim, E., Jiang, B., Goodman, M.T., Sibug-Saber, M., Cozen, W., et al. (2011). Human Papillomavirus and Rising Oropharyngeal Cancer Incidence in the United States. J. Clin. Oncol. *29*, 4294–4301.

7. Pytynia, K.B., Dahlstrom, K.R., and Sturgis, E.M. (2014). Epidemiology of HPV-associated oropharyngeal cancer. Oral Oncol. *50*, 380–386.

8. Haeggblom, L., Attoff, T., Yu, J., Holzhauser, S., Vlastos, A., Mirzae, L., Ährlund-Richter, A., Munck-Wikland, E., Marklund, L., Hammarstedt-Nordenvall, L., et al. (2019). Changes in incidence and prevalence of human papillomavirus in tonsillar and base of tongue cancer during 2000-2016 in the Stockholm region and Sweden. Head Neck *41*, 1583–1590.

9. PDQ® Adult Treatment Editorial Board (2024). PDQ Oropharyngeal Cancer Treatment (National Cancer Institute). https://www.cancer.gov/types/head-and-neck/hp/adult/oropharyngeal-treatment-pdq.

10. Licitra, L., Bernier, J., Grandi, C., Merlano, M., Bruzzi, P., and Lefebvre, J.-L. (2002). Cancer of the oropharynx. Crit. Rev. Oncol. Hematol. *41*, 107–122.

11. Lechner, M., Frampton, G.M., Fenton, T., Feber, A., Palmer, G., Jay, A., Pillay, N., Forster, M., Cronin, M.T., Lipson, D., et al. (2013). Targeted next-generation sequencing of head and neck squamous cell carcinoma identifies novel genetic alterations in HPV+ and HPV- tumors. Genome Med. *5*, 49.

12. Tinhofer, I., Budach, V., Saki, M., Konschak, R., Niehr, F., Jöhrens, K., Weichert, W., Linge, A., Lohaus, F., Krause, M., et al. (2016). Targeted next-generation sequencing of locally advanced squamous cell carcinomas of the head and neck reveals druggable targets for improving adjuvant chemoradiation. Eur. J. Cancer *57*, 78–86.

13. Bersani, C., Sivars, L., Haeggblom, L., DiLorenzo, S., Mints, M., Ährlund-Richter, A., Tertipis, N., Munck-Wikland, E., Näsman, A., Ramqvist, T., and Dalianis, T. (2017). Targeted sequencing of tonsillar and base of tongue cancer and human papillomavirus positive unknown primary of the head and neck reveals prognostic effects of mutated FGFR3. Oncotarget *8*, 35339–35350.

14. Beaty, B.T., Moon, D.H., Shen, C.J., Amdur, R.J., Weiss, J., Grilley-Olson, J., Patel, S., Zanation, A., Hackman, T.G., Thorp, B., et al. (2020). PIK3CA Mutation in HPV-Associated OPSCC Patients Receiving Deintensified Chemoradiation. J. Natl. Cancer Inst. *112*, 855–858.

15. Zupancic, M., Kostopoulou, O.N., Marklund, L., and Dalianis, T. (2025). Therapeutic options for human papillomavirus-positive tonsil and base of tongue cancer. J. Intern. Med. *297*, 608–629.

16. André, F., Mills, D., and Taran, T. (2019). Alpelisib for PIK3CA-Mutated Advanced Breast Cancer. Reply. N. Engl. J. Med. *381*, 687.

17. Bahleda, R., Italiano, A., Hierro, C., Mita, A., Cervantes, A., Chan, N., Awad, M., Calvo, E., Moreno, V., Govindan, R., et al. (2019). Multicenter Phase I Study of Erdafitinib (JNJ-42756493), Oral Pan-Fibroblast Growth Factor Receptor Inhibitor, in Patients with Advanced or Refractory Solid Tumors. Clin. Cancer Res. *25*, 4888–4897.

18. Holzhauser, S., Wild, N., Zupancic, M., Ursu, R.G., Bersani, C., Näsman, A., Kostopoulou, O.N., and Dalianis, T. (2021). Targeted Therapy With PI3K and FGFR Inhibitors on Human Papillomavirus Positive and Negative Tonsillar and Base of Tongue Cancer Lines With and Without Corresponding Mutations. Front. Oncol. *11*, 640490.

19. Kostopoulou, O.N., Zupancic, M., Pont, M., Papin, E., Lukoseviciute, M., Mikelarena, B.A., Holzhauser, S., and Dalianis, T. (2022). Targeted Therapy of HPV Positive and Negative Tonsillar Squamous Cell Carcinoma Cell Lines Reveals Synergy between CDK4/6, PI3K and Sometimes FGFR Inhibitors, but Rarely between PARP and WEE1 Inhibitors. Viruses *14*, 1372.

20. Lukoceviciute, M., Zupancic, M., Kostopoulou, O.N., Holzhauser, S., and Dalianis, T. (2024). Curcumin Alone and Combined With PI3K Inhibitors Elicits Positive Effects on Oropharyngeal Cancer Cell Lines Regardless of HPV Status. Anticancer Res. *44*, 1863–1876.

21. Birgersson, M., Chabanová, T., Wiechec, E., Kostopoulou, O.N., and Dalianis, T. (2025). Fibroblast Supernatants Modulate Treatment Responses in Human Papillomavirus Positive and Negative Oropharyngeal Cancer Cell Lines. Anticancer Res. *45*, 4729–4742.

22. Mehta, G., Hsiao, A.Y., Ingram, M., Luker, G.D., and Takayama, S. (2012). Opportunities and challenges for use of tumor spheroids as models to test drug delivery and efficacy. J. Contr. Release *164*, 192–204.

23. Nath, S., and Devi, G.R. (2016). Three-dimensional culture systems in cancer research: Focus on tumor spheroid model. Pharmacol. Ther. *163*, 94–108.

24. Fontoura, J.C., Viezzer, C., dos Santos, F.G., Ligabue, R.A., Weinlich, R., Puga, R.D., Antonow, D., Severino, P., and Bonorino, C. (2020). Comparison of 2D and 3D cell culture models for cell growth, gene expression and drug resistance. Mater. Sci. Eng., C *107*, 110264.

25. Lukoseviciute, M., Birgersson, M., Ceriani, P., Wilhelm, M., and Kostopoulou, O.N. (2026). Dual PI3K/AKT and CDK4/6 inhibition reveals selective sensitivity in an SHH medulloblastoma stem cell model. Mol. Oncol. https://doi.org/10.1002/1878-0261.70230.

26. Pirotte, E.F., Holzhauser, S., Owens, D., Quine, S., Al-Hussaini, A., Christian, A.D., Giles, P.J., Man, S.T., Evans, M., and Powell, N.G. (2018). Sensitivity to inhibition of DNA repair by Olaparib in novel oropharyngeal cancer cell lines infected with Human Papillomavirus. PLoS One *13*, e0207934.

27. Holzhauser, S., Pirotte, E., Jones, J., Owens, D., Al-Hussaini, A., Giles, P., Evans, M., Man, S., and Powell, N. (2020). Sensitivity of human papillomavirus-positive and -negative oropharyngeal cancer cell lines to ionizing irradiation. Oncol. Rep. *44*, 1717–1726.

28. Zheng, S., Wang, W., Aldahdooh, J., Malyutina, A., Shadbahr, T., Tanoli, Z., Pessia, A., and Tang, J. (2022). SynergyFinder Plus: Toward Better Interpretation and Annotation of Drug Combination Screening Datasets. Genom. Proteom. Bioinform. *20*, 587–596.

OMTON, Volume *34*

Supplemental information

Dual targeting of PI3K and FGFR or CDK4/6 reveals synergistic responses in HPV-positive and HPV-negative oropharyngeal cancer spheroids

Madeleine Birgersson, Monika Lukoseviciute, Ourania N. Kostopoulou, and Tina Dalianis

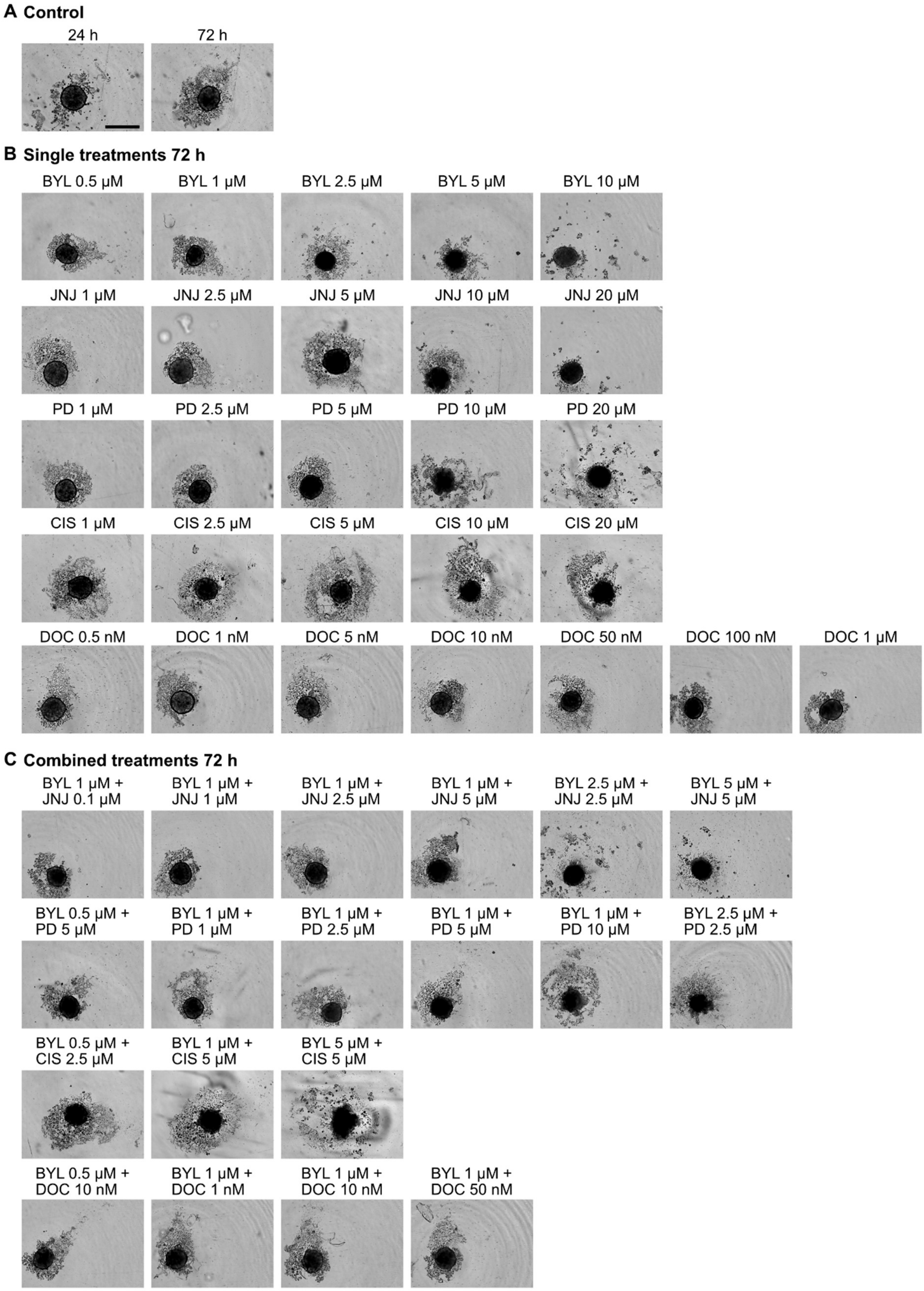

Figure S1. Effects of single PI3K, FGFR and CDK4/6 inhibitors and chemotherapeutic agents, and their combination with a PI3K inhibitor, on CU-OP-20 spheroid growth. Spheroid growth was monitored using the

Incucyte® SX5 Live-Cell Analysis Instrument from Sartorius. Representative images at 72h after treatment with: (A) PBS (control), (B) single treatment with BYL719, JNJ-42756493, PD-0332991, cisplatin, docetaxel, and (C) their combination with BYL719. The scale bar represents 400 µm.

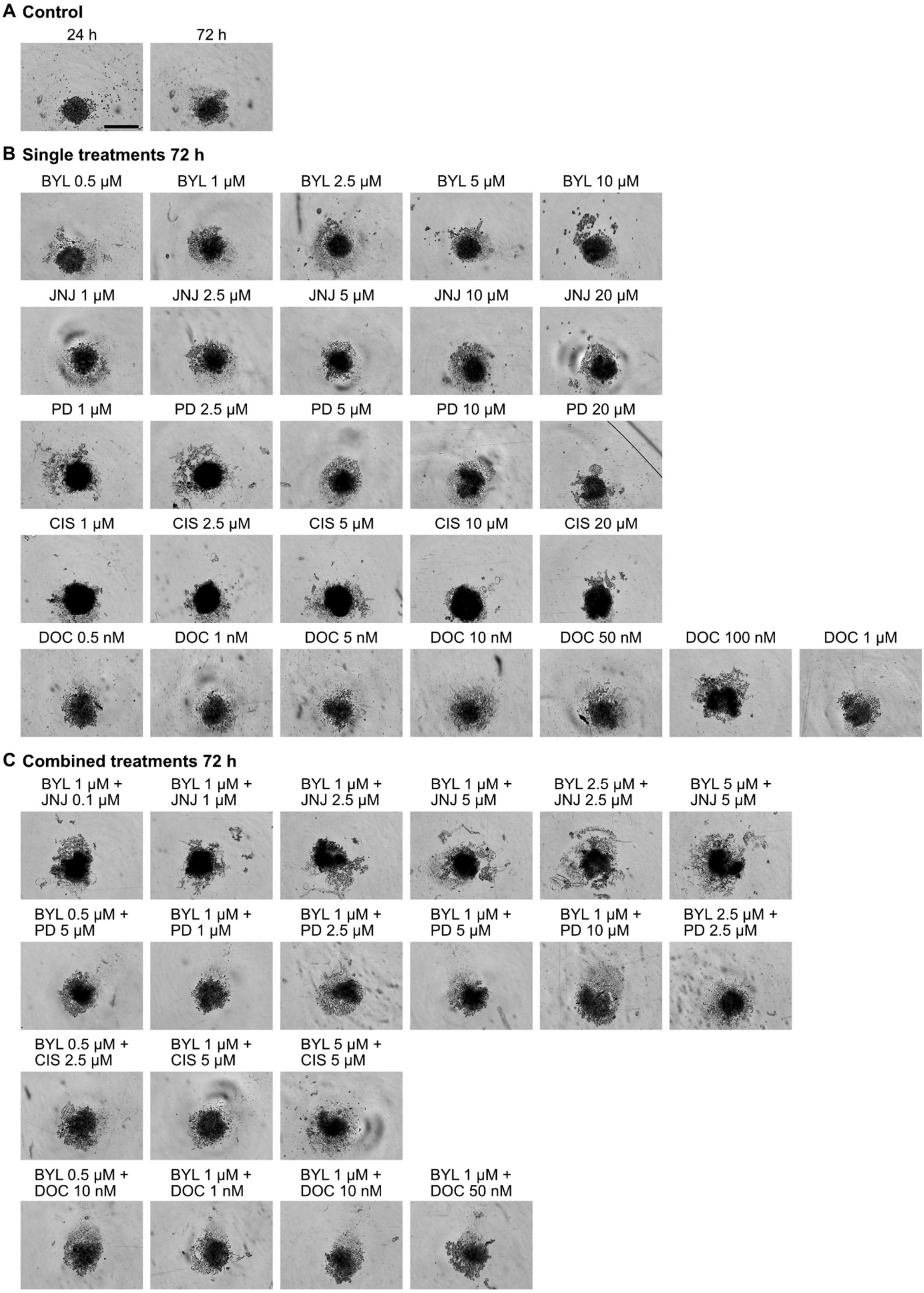

Figure S2. Effects of single PI3K, FGFR and CDK4/6 inhibitors and chemotherapeutic agents, and their combination with a PI3K inhibitor, on CU-OP-17 spheroid growth. Spheroid growth was monitored using

the Incucyte® SX5 Live-Cell Analysis Instrument from Sartorius. Representative images at 72h after treatment with: (A) PBS (control), (B) single treatment with BYL719, JNJ-42756493, PD-0332991, cisplatin, docetaxel, and (C) their combination with BYL719. The scale bar represents 400 μm.
